# Bilateral frosted branch angiitis in an initial case of systemic lupus erythematosus

**DOI:** 10.5935/0004-2749.20210074

**Published:** 2021

**Authors:** Caroline Oliveira Brêtas, Andreia Novelli, Thiago George Cabral Silva, Ledilma Inês Colodetti Zanandrea, Patrícia Grativol Costa Saraiva, Manuele Martins Vieira, Fábio Petersen Saraiva

**Affiliations:** 1 Departamento de Oftalmologia, Medicina Especializada, Centro de Ciências da Saúde, Universidade Federal do Espírito, Vitória, ES, Brazil; 2 Centro de Ciências da Saúde, Universidade Federal do Espírito, Vitória, ES, Brazil

**Keywords:** Lupus erythematosus, systemic/complications, Retinal vasculitis/etiology, Retinal vasculitis/drug therapy, Immunosuppressive agents/therapeutic use, Case reports, Lupus eritematoso sistêmico/complicações, Vasculite retiniana/etiologia, Vasculite retiniana/tratamento farmacológico, Imunossupressores/uso terapêutico, Relato de casos

## Abstract

Frosted branch angiitis is a rare and severe form of retinal vasculitis. It may be idiopathic or arise secondary to a systemic disease. We have reported here an unusually severe case of frosted branch angiitis in a previously healthy 13-year old girl who presented with significantly reduced vision in both eyes. In this case, frosted branch angiitis was one of the presentations of systemic lupus erythematosus. The characteristic patterns of frosted branch angiitis were observed on fundoscopy in both eyes. An extensive etiological study was conducted, whereby the diagnosis of systemic lupus erythematosus was confirmed. Only a few such cases have been reported so far in the literature.

## Abstract (pt)

A angeíte congelada é tipo específico de vasculite retiniana rara e grave. Pode ser idiopática ou secundária à doença sistêmica. Relatamos um incomum caso com associação ao lúpus eritematoso sistêmico, sendo a angeíte congelada uma das manifestações do quadro. Uma jovem de 13 anos, previamente hígida, apresentou queixa de baixa visual importante bilateral. À fundoscopia foi evidenciado o aspecto de angeíte de vasos congelados em ambos os olhos. Extensa investigação etiológica foi realizada com diagnóstico confirmado de lúpus eritematoso sistêmico. Poucos casos foram descritos na literatura.

## INTRODUCTION

Frosted branch angiitis (FBA) is a rare form of retinal vasculitis that is characterized by the presentation of severe diffuse lymphoplasmacytic infiltration of the perivascular space^(1)^. FBA may be idiopathic or arise secondary to autoimmune disorders, viral infections, or malignancies^(2)^. Clinically, it manifests as rapid visual deterioration, diffuse vascular sheathing, macular edema, papillitis, vitreitis, and anterior uveitis^(2)^. The involvement of the retina is rarely complete. However, in this paper, we have described the case of a girl with bilateral FBA that affected her entire retina as one of the presentations of systemic lupus erythematosus (SLE).

## CASE REPORT

A previously healthy 13-year old girl was hospitalized with a history of high fever and unspecific symptoms for 20 days. She denied signs of skin rash, oral or nasal ulcers, joint symptoms, weakness, dyspnea, or lymphadenomegaly. She had received the medication of ciprofloxacin and azithromycin with no response. The patient was therefore admitted with the following normal routine examination reports: myelogram, bilirubin (total, direct, and indirect), transaminases, alkaline phosphatase, urea, creatinine, lactate dehydrogenase, uric acid, thyroid stimulating hormone and total complement (CH50), and fractions (C3 and C4). The level of fibrinogen, ferritin, erythrocyte sedimentation rate (ESR), C-reactive protein and procalcitonin demonstrated a dosage higher than the normal, which suggested inflammatory activity. The results of prothrombin time, activated partial thromboplastin time, and 24h proteinuria were altered and the blood count indicated pancitopenia. The findings of abdominal ultrasound and echocardiogram were suggestive of serositis.

**Figure 1:**
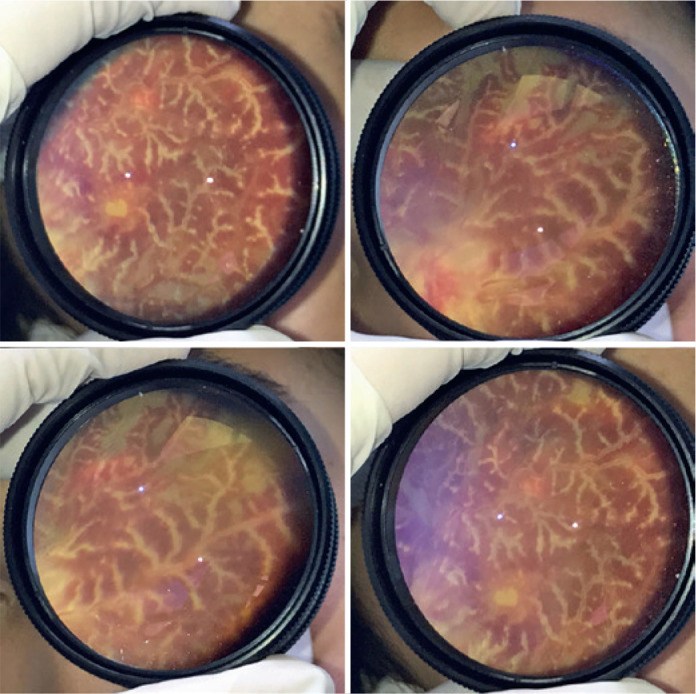
Fundoscopy of both eyes at the time of diagnosis of Frosted

The patient was accordingly started on ceftriaxone. On the fifth day of hospitalization, the patient woke up with headache, vomiting and complaints of severely reduced visual acuity in both her eyes. A lumbar biopsy revealed presence of turbid inflammatory fluid. The ophthalmological findings were visual acuity of hand motion in both eyes and the fundoscopy revealed exudates, diffuse hemorrhages and vascular sheathing affecting the entire retina, all of which confirmed the diagnosis of FBA in both eyes (Figure 1). Cranial magnetic resonance angiography revealed areas of signal alteration in the thalamus and corpus callosum with the evidence of retinal and cerebral vasculitis.

The patient was antinuclear factor-positive (ANA), with a homogenous nuclear pattern at a dilution of 1/640. All the other tests conducted were negative, including antiphospholipid antibodies, Anti-RNP, Anti-Ro, Anti-La, Anti-SM, Direct Coombs, Venereal Disease Research Laboratory, serology for dengue, toxoplasmosis, mycoplasma and hepatitis. Serology for Epstein-Barr (EBV), herpes and cytomegalovirus (CMV) were positive for IgG.

Based on these findings, the patient was referred to a rheumatologist and subsequently diagnosed with severe SLE based on the following diagnostic criteria: non-hemolytic anemia, thrombocytopenia, serositis, altered 24h proteinuria, retinal vasculitis and positive ANA. The patient was prescribed with i.v. immunoglobulin for 2 days and initiated on pulse therapy with methylprednisolone and cyclophosphamide for 3 days. After 21 days, the patient was discharged with a prescription of prednisone at the dosage of 40 mg/day. Changes in her blood count, inflammatory tests, coagulation and cranial magnetic resonance imaging suggested improvement during the follow-up period.

**Figure 2:**
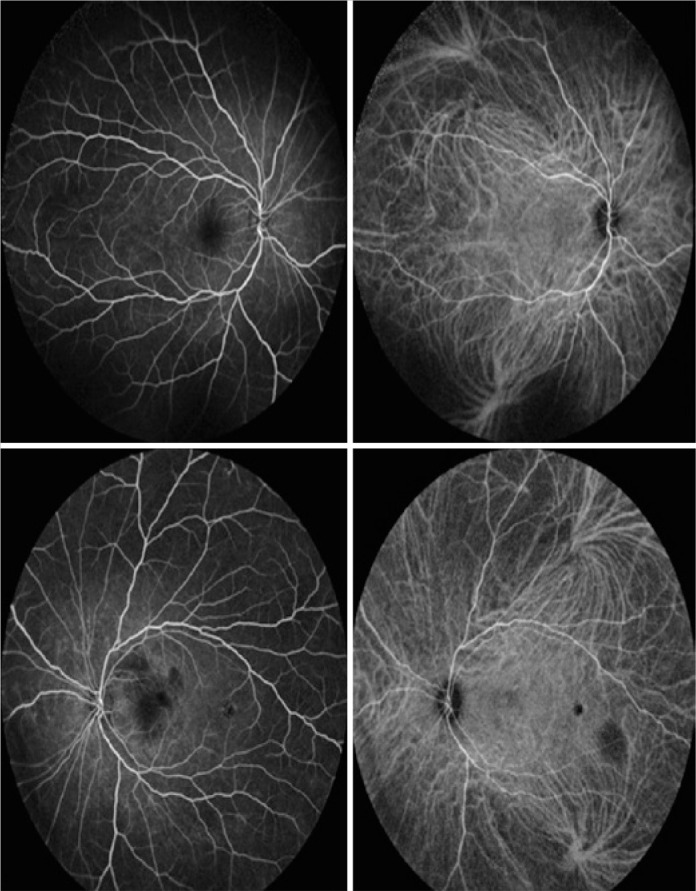
Fluorescein angiography and indocyanine green angiography branch angiitis demonstrating aggressive and diffuse vascular sheathing, demonstrating hypofluorescent areas in both eyes at 2 months after the hemorrhage of the entire retina, and severe local edema. treatment.

**Figure 3:**
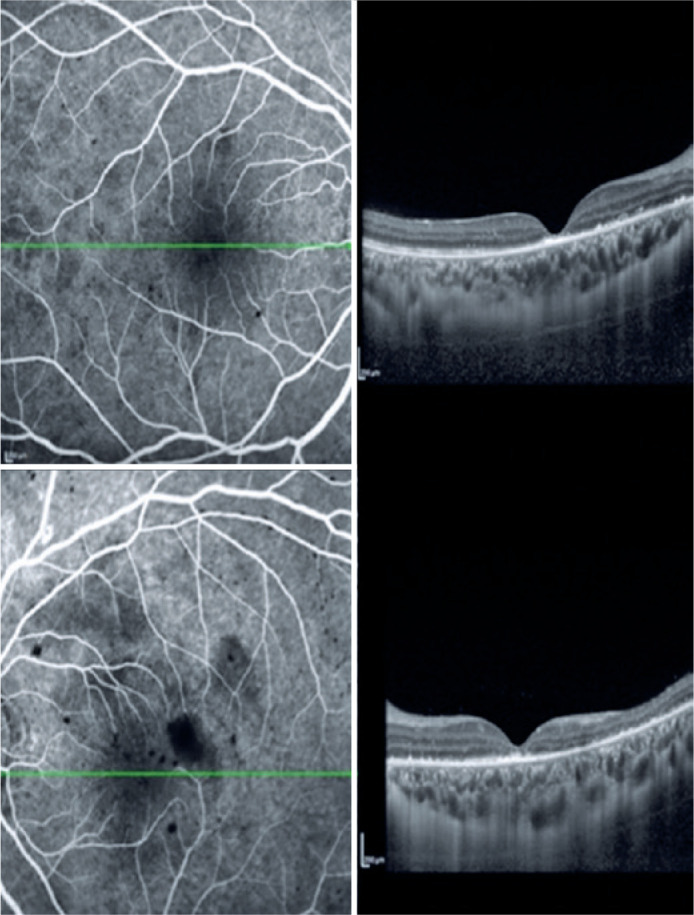
Optic coherence tomography of both eyes 2 months after the treatment showing hyper-reflectivity in the outer retinal layers (suggestive of foveal atrophy).

**Figure 4:**
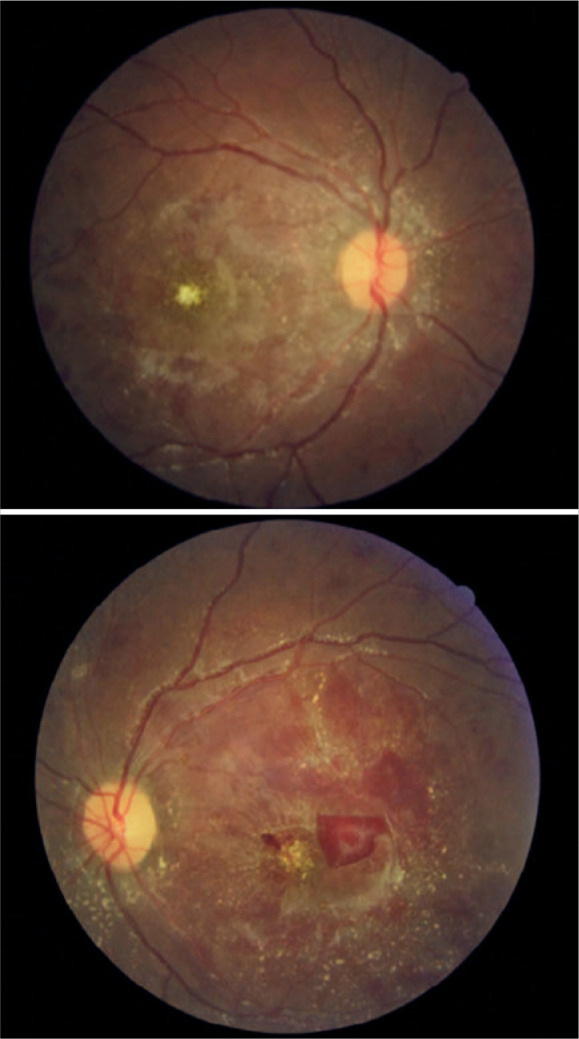
Retinography of both eyes 2 months after the treatment demonstrating improvement in the ophthalmological conditions, despite the persistence of exudations and hemorrhages.

After 2 months, the patient returned for a multimodal study of the retina, which revealed bilateral foveal atrophy of the entire retina and significant temporal inner retinal thinning in the left eye (Figures 2, 3 and 4). It was not possible to perform the multimodal analysis at the onset of the ocular condition as the patient was hospitalized in an intensive care unit in a serious condition, which made her displacement impossible. After 4 months, the patient’s best-corrected visual acuity was 20/50 in the right eye and 20/200 in the left eye. At this time point, no changes were observed on biomicroscopy, while the fundoscopy revealed mild optic disc pallor, preserved vessels without hemorrhage, dry macula, heterogeneous retina color, and clear vitreous body.

## DISCUSSION

FBA was first described by Japanese researchers in 1976 when reporting the case of a 6-year-old boy with severe sheathing of the retinal vessels, that resembled the frosted tree branches^(3)^. The prevalence of FBA is higher among women. The condition peaks bimodally at 6 and 16 years of age in Japanese reports and in the 30s elsewhere^(3)^. FBA is usually bilateral^(3)^.

In 1997, Kleiner proposed the classification of FBA into 3 types. The first type is associated with leukemia and lymphoma, and vascular sheathing is attributed to malignant cell infiltration into the vascular walls, with no inflammatory process. The second type is true vasculitis secondary to infection or active autoimmune diseases^(4)^. The third type is idiopathic and affects healthy young individuals with a history of infection capable of inducing ocular inflammation^(4)^. According to Ito et al., until 2004, only 57 cases of FBA had been reported, most of which were from Japan (75%)^(3)^. Moreover, very few reports indicated the association between FBA and SLE^(5)^.

SLE is classified into 2 groups: the Systemic Lupus International Collaborating Clinics criteria (SLICC, 2012) and the American College of Rheumatology (ACR, 1997). In this case, we selected the SLICC because it was more recent and showed a greater sensitivity in both the adult and pediatric populations^(6)^. This system included the following clinical criteria: acute cutaneous lupus, chronic cutaneous lupus, non-scarring alopecia, oral or nasal ulcers, synovitis involving ≥2 joints with at least 30 min of morning stiffness or swelling or effusion, serositis, renal involvement (altered protein/creatinine ratio, 24h proteinuria>500 mg or red blood cell casts), hemolytic anemia, leukopenia (<4000), lymphopenia (<1000), and thrombocytopenia (<100000). The immunological criteria included the presence of antinuclear antibodies (ANA), Anti-dsDNA, Anti-Sm, Antiphospholipid, low complement (C3, C4, or CH50) and the Direct Coombs test in the absence of hemolytic anemia^(6)^.

To diagnose SLE, SLICC requires at least 4 criteria with at least 1 clinician and 1 immunological or lupus nephritis as the only clinical criterion in the presence of ANA or Anti-dsDNA^(6)^. The patient showed non-hemolytic anemia, thrombocytopenia, serositis, altered 24h proteinuria, retinal vasculitis, and positive ANA. In the present case, the clinical presentation was particularly exuberant as the entire retina was compromised in both the eyes, rather than in specific sectors and vascular branches.

Retinopathy is a major manifestation of SLE that affects 12%-26% of the patients^(7)^. However, the most frequently observed fundoscopic change was microangiopathy (30%), which was characterized by cotton-wool spots without or with associated intraretinal hemorrhage^(8,9)^. FBA secondary to SLE was an uncommon form that reflected intense disease activity and, more rarely, the initial symptom. Only a small number of cases have been described so far, with varying signs and symptoms^(5,9,10)^. The pathogenesis of this type of FBA involves the presence and deposition of immune complexes (IC) in the vessel walls^(5,9,10)^.

Circulating IC are responsible for activating the complement cascade (such as classical and alternative pathways) and alteration of fragment crystallizable (Fc receptors) function of the immune cells^(11)^. The activated complement binds to the IC and allows its clearance, avoiding deposition in inappropriate places such as the kidney and vascular endothelium^(5,11)^. This clearance is possible due to the binding of the complex IC-complement proteins to the CR1 receptor of erythrocytes, which allows the transport to the macrophages and their destruction^(5)^. The failure of these mechanisms leads to the deposition of IC in the tissues, sustaining an inflammatory process and the consequent damages^(5)^. Anemia and consumption of complement, which may or may not be present in SLE, are used for the evaluation of therapeutic response^(5)^.

Past reports have shown association between autoimmune diseases with infections, and these relations can be protective or causative. According to the literature, some cases of SLE occur in concomitance with EBV, parvovirus B19, retrovirus, and/or CMV, and it is suggested that these agents activate the autoimmunity mechanisms^(12)^.

Our patient showed bilateral FBA as one of the signs of SLE. Although this condition is rare, we believe that the ophthalmological involvement was mainly resultant from an exacerbation of the underlying disease, with vasculitis secondary to the deposition of IC. The patient’s rapid and positive response to systemic corticoid therapy suggests a relevant immune component. Furthermore, the presence of positive serology for EBV, CMV, and herpes revealed its influence in the pathophysiology of SLE. The present report also highlights the importance of systemic investigations in patients with retinal vasculitis, considering its severity and the potential sequelae.
